# Antioxidant Activity of Papaya Seed Extracts

**DOI:** 10.3390/molecules16086179

**Published:** 2011-07-25

**Authors:** Kaibing Zhou, Hui Wang, Wenli Mei, Xiaona Li, Ying Luo, Haofu Dai

**Affiliations:** 1 Key Laboratory of Protection and Development Utilization of Tropical Crop Germplasm Resources (Hainan University), Ministry of Education, Haikou 570228, China; 2 The Institute of Tropical Bioscience & Biotechnology, Chinese Academy of Tropical Agriculture Sciences, Haikou 571101, China

**Keywords:** papaya, seeds, antioxidant activity, radical scavenging activities

## Abstract

The antioxidant activities of the ethanol, petroleum ether, ethyl acetate, *n*-butanol and water extract fractions from the seeds of papaya were evaluated in this study. The ethyl acetate fraction showed the strongest DPPH and hydroxyl free radical-scavenging activities, and its activities were stronger than those of ascorbic acid and sodium benzoate, respectively. The *n*-butanol fraction demonstrated the greatest ABTS^+^ radicals scavenging activity. The ethyl acetate fraction and the *n*-butanol fraction not only showed higher antioxidant activities than the petroleum ether fraction, water fraction and ethanol fraction, but also showed higher superoxide anion and hydrogen peroxide radicals scavenging activities than those of the other extract fractions. The high amount of total phenolics and total flavonoids in the ethyl acetate and *n*-butanol fractions contributed to their antioxidant activities. The ethyl acetate fraction was subjected to column chromatography, to yield two phenolic compounds, *p*-hydroxybenzoic acid (**1**) and vanillic acid (**2**), which possessed significant antioxidant activities. Therefore, the seeds of papaya and these compounds might be used as natural antioxidants.

## 1. Introduction

Papaya (*Carica papaya* L.), a kind of tropical evergreen fruit tree originated from Mexico and Central America, is mainly found distributed in the south of China, such as Hainan, Guangdong, Guangxi, Yunnan, Taiwan, and Fujian Province. Hainan Province is the optimum region to cultivate papaya in China. Much peel and seeds waste is produced after the processing and consumption of papaya fruits. This waste, that usually polluted our habitat, could actually be utilized. Philippine ethnomedical information on papaya revealed that the fruits, stems, leaves, and roots may be used as anthelmintics, stomachic, antidyseptic, diuretics, emmenagogue, laxative, vermifuge, antiasthmatic, antirheumatic, rubefacient, tonic, poultice, and as a cure for enlargement of liver, spleen, freckles, and cancerous growths [1,2]. The fruits and the waste of papaya have been utilized as a new medicine as well as invigorant and cosmetic in recent years in China.

Much attention had been paid to the abundance of papain and lipase of papaya in all organs, and some scholars thought these two enzymes contributed to some of the functions of papaya mentioned above [3,4,5]. Some functions of papaya were related to the antioxidant activity of some secondary metabolites in the papaya organs. Early studies on the DPPH, hydroxyl, and superoxide free radical-scavenging activities of some tropical fruits and the water extract fraction from the flesh seeds of papaya indicated that it exhibited the strongest activities [6,7,8,9]. Up to now, the antioxidant activities of the other extract fractions from papaya seeds have not been studied, so it was necessary to study their antioxidant activities for the purpose of the evaluating the potential utilization of this waste.

Each method used for testing the antioxidant activities of natural medicine and foods *in vitro* had its limitations, so several methods were always used together to identify the antioxidant activities of natural products [10]. In this paper, six different methods were used to evaluate the antioxidant activities of the different solvent fractions obtained from papaya seeds.

## 2. Results and Discussion

### 2.1. DPPH Radical Scavenging Assay 

**Figure 1:**
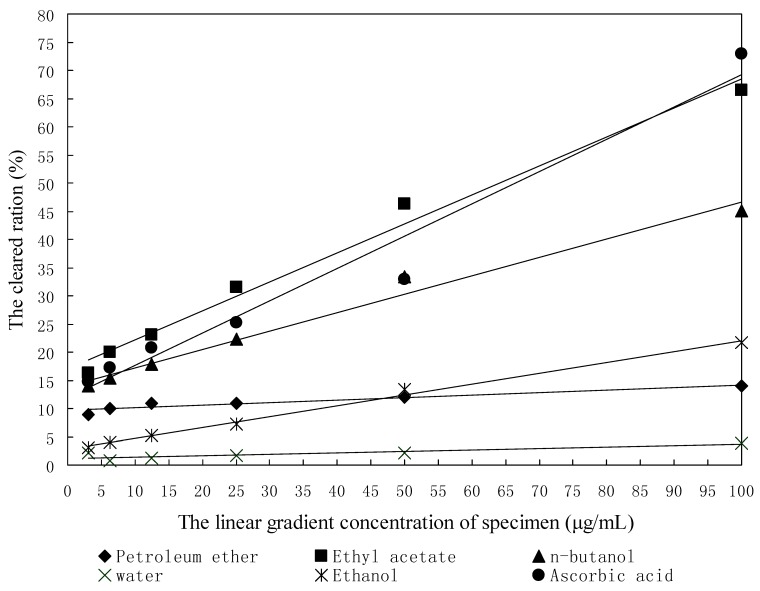
The regression curves of DPPH.

The DPPH free radical-scavenging activities of the five studied samples were estimated by comparing the EC_50_ of the extract fractions and ascorbic acid. It was found that the radical-scavenging activities of the positive control and various solvent extract increased with increasing concentration, and all the regression equations were significant at p < 0.05 (Figure 1 and Table 1), so all five of the studied samples had DPPH free radical-scavenging activity. According to the EC_50_ values, the ability to scavenge DPPH free radicals of the five studied samples could be ranked as ethyl acetate fraction > ascorbic acid > *n*-butanol fraction > ethanol fraction > petroleum ether fraction > water fraction. The EC_50_ values of DPPH free radical-scavenging activities of the ethyl acetate fraction, *n*-butanol fraction, ethanol fraction, petroleum ether fraction, water fraction and ascorbic acid were found to be 64.61 μg/mL, 109.30 μg/mL, 248.63 μg/mL, 1,009.50 μg/mL, 1,628.33 μg/mL and 66.96 μg/mL, respectively. So the DPPH free radical-scavenging activity of the ethyl acetate fraction indicated the strongest antioxidant activity, and the *n*-butanol fraction had stronger antioxidant activity too. An almost linear correlation between DPPH free radical-scavenging activity and concentrations of polyphenolic compounds in various vegetables and fruits have been reported [11]. This indicated that DPPH free radical-scavenging activities of all extracts from seeds of papaya were related to the amount of antioxidant constituents extracted from seeds of papaya by various solvents. These results also revealed that the ethyl acetate fraction and the *n*-butanol fraction from papaya seeds contained free radical scavengers, acting possibly as primary antioxidants.

### 2.2. Total Antioxidant Capacity by Trolox Equivalent Antioxidant Capacity (TEAC) Assay

The TEAC value is a quantification of the effective antioxidant activity of the extracts. The higher TEAC value implies a greater antioxidant activity. Total antioxidant potential of the extracts defined as the amount of Trolox with the equivalent antioxidant activity as 1 g dry weight of the tested substances. The ABTS ^+^ scavenging activities of the five studied samples are shown in Table 2. The ABTS ^+^ scavenging activity of the *n*-butanol fraction was significantly stronger than that of the others. There was an insignificant difference in the ABTS ^+^ scavenging activities between the ethanol fraction and the ethyl acetate fraction. The ABTS ^+^ radical-scavenging activity of the petroleum ether fraction was significantly stronger than that of the water fraction, and extremely worse than that of the others. The ABTS·^+^ radical-scavenging activity of the water fraction was the poorest. It has been reported that flavonoids with efficient scavenging properties have a TEAC value of ≥1.9 mM, in comparison to less efficient antioxidants with a TEAC value of ≤1.5 mM [12]. By this criteria the ethyl acetate fraction, the *n*-butanol fraction and the ethanol fraction might function as an efficient antioxidant according to the TEAC values listed in Table 2.

### 2.3. Determination by FRAP Assay

In this assay, extracts and fractions were used in a redox-linked reaction whereby the antioxidants present in the sample act as the oxidants. Reduction of the ferric-tripyridyltriazine complex to the ferrous complex forms an intense blue colour which can be measured at 593 nm. The intensity of the colour is related to the amount of antioxidant reductants in the extracts. The trend for ferric ion-reducing activities of different fractions is shown in Table 2. The antioxidant activity of the ethyl acetate fraction was significantly stronger than that of the others. There was insignificant difference in the antioxidant activities among the ethanol extract fraction, *n*-butanol fraction and water fraction, while the antioxidant activity of the petroleum ether fraction was the poorest. Like the results obtained from the DPPH and ABTS assay, the ethyl acetate fraction showed relatively strong ferric ion-reducing activity while all the other fractions showed lower ferric ion-reducing activities.

### 2.4. The Superoxide Anion Radical-Scavenging Activity 

Superoxide anion radical is an initial radical and plays an important role in the formation of other reactive oxygen-species such as hydroxyl radical, hydrogen peroxide, or singlet oxygen in living systems [13], so superoxide radical is known to be very harmful to cellular components, contributing to tissue damage and various diseases [14]. Table 2 shows the superoxide anion radical-scavenging activities of the five studied samples. There was no significant difference in the superoxide anion radical-scavenging activities between the *n*-butanol fraction and ethyl acetate fraction. The superoxide anion radical-scavenging activities of the ethyl acetate fraction and *n*-butanol fraction were significantly stronger than those of the ethanol fraction and the other two fractions. The results suggested that the extracts displayed scavenging effect on superoxide anion radical generation that could prevent ameliorate oxidative damage.

### 2.5. The Hydrogen Peroxide Radical-Scavenging Activity 

Biological systems can produce hydrogen peroxide. Hydrogen peroxide itself is not very active, but it can sometimes be toxic to cells, since it may give rise to hydroxyl radicals inside the cell [15]. Hydrogen peroxide also can attack many cellular energy-producing systems. For instance, it deactivates the glycolytic enzyme glyceraldehyde-3-phosphate dehydrogenase [16]. The hydrogen peroxide radical-scavenging activities of the five studied samples were shown in Table 2. There was no significant difference between the ethyl acetate fraction and the *n*-butanol fraction, also between the ethyl acetate fraction and the petroleum ether fraction, but there was extremely significant difference between the *n*-butanol fraction and the petroleum ether fraction. There were extremely significant differences in the hydrogen peroxide radical-scavenging activities among other extracts. In summary, the hydrogen peroxide radical-scavenging activities were poorer and the order was the *n*-butanol fraction and the ethyl acetate fraction > petroleum ether fraction > ethanol fraction > water fraction.

### 2.6. The Hydroxyl Radical-Scavenging Activity

**Figure 2:**
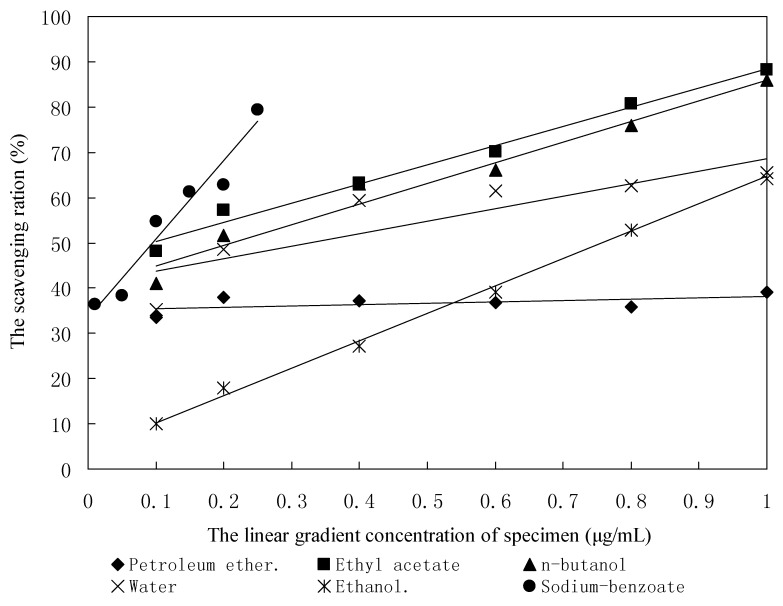
The regression lines of the scavenging abilities of OH.

Among the oxygen radicals, hydroxyl radical is the most active and induces severe damage to adjacent biomolecules [17]. In this study, the Fenton reagent (Fe^2+^ + H_2_O_2_ Fe^3+^ +OH^−^ + ^−^OH) as a source of hydroxyl radical was used to test the scavenging activity of the five studied samples towards hydroxyl radical. As shown in Figure 2 and Table 3, all five studied samples except for the petroleum ether fraction exhibited potent or moderate activity in an concentration dependent manner. The linear regression equations of the hydroxyl radical-scavenging activities on the concentrations of sodium benzoate and the five studied samples except the petroleum ether fraction were significant at the significance level of 0.05. It indicated the five studied samples except for the petroleum ether fraction had hydroxyl radical-scavenging activity. According to the EC_50_ in Table 3, the hydroxyl radical-scavenging activity of the ethyl acetate fraction was slightly stronger than that of sodium benzoate. The EC_50_ values of hydroxyl radical-scavenging activity of the *n*-butanol fraction, water fraction, ethanol fraction and sodium benzoate were found to be 0.21 μg/mL, 0.33 μg/mL, 0.76 μg/mL and 0.09 μg/mL, respectively.

### 2.7. Total Phenolics

The Folin–Ciocalteu assay is a fast and simple method to rapidly determine the amount of phenolic compounds in samples. Phenols or polyphenols are secondary metabolites that are present in every plant and plant products. Phenolic compounds contribute to the overall antioxidant activities of plants. Generally, the mechanisms of phenolic compounds for antioxidant activity are inactivating lipid free radicals and preventing decomposition of hydroperoxides into free radicals. Kumar *et al*. found that gallic acid and tannic acid, in the phenolic fraction, are the major antioxidant compounds of *Phyllanthus emblica* [18]. Jeong *et al*. also found that the antioxidant activity of the *n*-butanol fraction from the aerial parts of *Platycodon grandiflorum* was attributable to some phenolic compounds such as luteolin-7-O-glucoside and apigenin-7-O-glucoside [19]. In this paper, the total phenolics of five fractions from papaya seeds are presented in Table 4. One-way ANOVA showed significant differences in total phenolic compounds content among the five studied samples. The ethyl acetate fraction exhibited the highest total phenolics, approximately 72-fold more than the ethanol fraction, 134-fold more than the *n*-butanol fraction, 272-fold more than the petroleum ether fraction and 603-fold more than the water fraction, respectively. Some authors have reported similar correlations between polyphenols and antioxidant activity measured by various methods [20]. A strong correlation between the mean values of the total polyphenol content and FRAP deserves detailed attention, because it implied that polyphenols in papaya seeds were capable of reducing ferric ions.

### 2.8. Total Flavonoids

The amount of the total flavonoids in the five studied samples are shown in Table 4. One-way ANOVA showed extremely significant differences in total flavonoids among the samples. The highest amount of total flavonoids was found in the ethyl acetate fraction, followed by *n*-butanol fraction, ethanol fraction and water fraction, while the content of total flavonoids couldn’t be detected in the petroleum ether fraction. It could be found that this order was similar to that of their DPPH radical-scavenging activities. Several reports had conclusively shown close relationship between antioxidant activity and the amount of total flavonoids [21,22,23]. Results in our study also showed that the extent of antioxidant activity of papaya seeds was in accordance with the amount of flavonoids present in this species.

The order of the antioxidant activites was similar to that of the amount of total phenolics and total flavonoids among the extract fractions. So the monomer compounds with antioxidant activities from these two fractions were deemed worth isolating. The ethyl acetate fraction and *n*-butanol fraction came from papaya whose fruits were edible, so these natural products from papaya seeds might be nontoxic and harmless to human, and they could be utilized as natural antioxidant of some products such as food and medicine.

### 2.9. Structural Elucidation of Isolated Compounds

**Figure 3:**
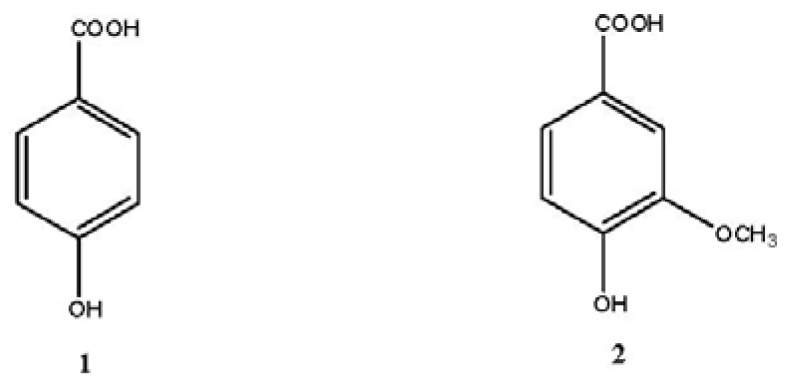
Structures of compounds **1**-**2**.

The structures of the isolated compounds were indentified on the basis of spectroscopic analyses including ^1^H- and ^13^C-NMR (DEPT) spectroscopy, combined with comparison of its NMR data to those reported in the literatures. The chemical structures of these isolates were identified as shown in Figure 3. *p*-hydroxybenzoic acid (**1**) and vanillic acid (**2**) are simple phenols. All the compounds were isolated from the seeds of papaya for the first time.

## 3. Experimental

### 3.1. Materials and Chemicals

The seeds of papaya were collected from Hainan Lvchao Biotechnical limited company in April, 2009. ^1^H- and ^13^C-NMR spectra were obtained using a Bruker AV-400 instrument using deuterated dimethyl sulfoxide (DMSO-*d*_6_), chloroform (CDCl_3_), acetone (CD_3_COCD_3_) and methanol (CD_3_OD) as solvents. Column chromatography was carried out on silica gel (200–300 mesh, Qingdao Marine Chemistry Company, Qingdao, China) and Sephadex LH-20 (Merck, Darmstadt, Germany). Optical density measurements were made with a Shimadzu UV-2550 spectrophotometer (Shimadzu, Kyoto, Japan). Folin-Ciocalteu’s reagent, 1,1-diphenyl-2-picrylhydrazyl (DPPH), 2,2’-azino-bis(3-ethylbenzothiazline-6-sulphonic acid) diammomium salt (ABTS), potassium persulfate, rutin, 6-hydroxy-2,5,7,8-tetramethychroman-2-carboxylic acid (Trolox), ferric chloride, sodium acetate, 2,4,6-tripyridyl-S-triazine (TPTZ), 2-thiobarbituric acid (TBA), ascorbic acid, xanthine, xanthine oxidase (XOD), α-tocopherol. All solvents were of analytical grade and purchased from Sigma Chemical Co. (St. Louis, MO, USA).

### 3.2. Extraction from Papaya Seeds and Isolation of Antioxidant Compounds

The dried and crushed papaya seeds (130.0 kg) were exhaustively extracted three times with 95% ethanol (90 L) at room temperature for three weeks. The ethanol extract was then filtered through absorbent gauze, and the filtrate was concentrated under reduced pressure to remove ethanol. The extract was suspended in H_2_O and partitioned with petroleum ether, ethyl acetate and *n*-butanol successively. All the extracts and aqueous layer were separately combined and evaporated to dryness under reduced pressure to yield petroleum ether fraction (10.2 g), ethyl acetate fraction (1.2 g), ***n***-butanol fraction (1.1 g), and water fraction (6.4 g), respectively. The ethyl acetate fraction fraction (1.2 g) was subjected to column chromatography (CC) over silica gel eluted with increasing polarities of a mixture of chloroform and methanol resulting in 5 fractions (Fr.1–Fr.11). Repeated CC on silica gel CC eluted with petroleum ether–acetone gradients (10:1–2:1, v/v) and Sephadex LH–20 (CHCl_3_–MeOH, 1:1, v/v) led to the isolation of compounds **1** (500.0 mg) and **2** (400.2 mg).

*P*-hydroxybenzoic acid (**1**): C_7_H_6_O_3_, colorless needles, ^1^H NMR (CD_3_COCD_3_, 400 MHz), *δ* 7.90 (2H, d, *J* = 8.8 Hz, H-2,6), 6.91 (2H, d, *J* = 8.8 Hz, H-3,5); ^13^C NMR (CD_3_COCD_3_, 100 MHz), *δ* 123.6 (s, C-1), 133.7 (d, C-2,6), 116.9 (d, C-3,5), 163.6 (s, C-4), 168.6 (s, C-7). The above data were identical to those in the literature [24]. 

Vanillic acid (**2**): C_8_H_8_O_4_, yellow powder, ^1^H NMR (CD_3_COCD_3_, 400 MHz), 7.62 (1H, dd, *J* = 8.2, 1.7 Hz, H-6), 7.59 (1H, d, *J* = 1.7 Hz, H-2), 6.93 (1H, d, *J* = 8.2 Hz, H-5), 3.91 (3H, s, OCH_3_); ^13^C NMR (CD_3_COCD_3_, 100 MHz), *δ* 125.9 (s, C-1), 114.5 (d, C-2), 149.0 (s, C-3), 153.0 (s, C-4), 116.5 (d, C-5), 123.8 (d, C-6), 168.8 (s, C-7), 57.3 (OCH_3_). The above data were consistent with the literature data [25].

### 3.3. DPPH Free Radical-Scavenging Activity

The DPPH radical-scavenging capacity was measured using the method of Blois [26] with some modification. Two milliliter of an ethanol solution of DPPH (0.1 mM) was added to sample fractions (0.1 mL, 0.075–0.1 mg/mL) in DMSO at different concentrations. After gentle mixing and 30 min of reaction at room temperature, the absorbances of the resulting solutions were measured at 517 nm. Ascorbic acid was used as the positive control. The DPPH radical-scavenging capacity (%) was calculated as DPPH scavenging = [(control absorbance - extract absorbance)/(control absorbance)] × 100%.

### 3.4. Total Antioxidant Capacity by Trolox Equivalent Antioxidant Capacity (TEAC) Assay

The TEAC assays were carried using a modified method as described by Re *et al*. [27]. Potassium persulfate was added to 7 mM of ABTS^+^ and kept for 12–16 h at room temperature in dark. The ABTS^+^ solution was diluted with PBS (potassium phosphate-buffered saline, pH 7.4) to an absorbance of 0.70 ± 0.02 at 730 nm before analysis. ABTS^+^ solution (1.485 mL) was added to sample fractions (15 μL) in DMSO at different concentrations and mixed by hand for 20 s. The reaction mixture was kept at room temperature for 6 min, and the absorbance was recorded at 730 nm on a Shimadzu UV-2550 spectrophotometer. Trolox was used as the positive control. The TEAC of the sample was expressed as Trolox equivalent in millimolars per 1 g dry weight of extracts (mmol Trolox/g DW).

### 3.5. Antioxidant Activity by the Ferric Reducing/Antioxidant Power (FRAP) Assay

The FRAP assays were carried using a modified method as described by Benzie and Strain [28]. Briefly, the ferric reducing/antioxidant power (FRAP) reagent containing 2.5 mL of a 10 mM TPTZ solution in 40 mM HCl, and 2.5 mL of 20 mM FeCl_3_ and 2.5 mL of 0.3 mM acetate buffer at pH 3.6 was prepared freshly and warmed at 37 °C. Aliquots of 40 µL of 0.1 mg/mL sample supernatant were mixed with 0.2 mL of distilled water and 1.8 mL of FRAP reagent. After incubation at 37 °C for 30 min, the absorbance of the reaction mixture at 593 nm was measured. The 1.0 mM FeSO_4_ was used as the standard solution. The antioxidant activity of the sample by the FRAP assay was expressed as FeSO_4_ equivalent in μmol per 1 g dry weight of extracts (μmol FeSO_4_/g DW).

### 3.6. Superoxide Radical-Scavenging Activity

The tested method was optimized based on the method described by Sakanaka *et al*. [29]. One milliliter of 65 mM phosphate buffer solution (pH7.8), 0.1 mL of 7.5 mM xanthine solution, 0.1 mL of 10 mM Hydroxylammonium chloride solution, 0.1 mL of 0.1 mg/mL sample solution, 0.4 mL of redistilled water and 0.3 mL of 200 μg/mL protein xanthine oxidase solution were mixed in turns, then incubated at 25 °C for 20 min. The reactive liquid was sampled 0.5 mL, and added 0.5 mL of 19 mM anhydrous *p*-aminobenzenesulfonic acid and 0.5 mL of 1.0% α-naphthylamine solution and mixed fully, then reacted at room temperature for 20 min. The absorbency (A_1_) was tested at 530 nm. The sample was substituted by redistilled water and repeated the procedures mentioned above to test the absorbency (A_0_) of the blank. The samples were substituted by the linear gradient concentrations of α-tocopherol to establish the standard curve. The superoxide radical-scavenging activity was shown with α-tocopherol equivalent antioxidant capacity (μmol α-tocopherol/g DW).

### 3.7. Hydrogen Peroxide Radica-Scavenging Activity

The tested method was optimized based on the method described by Patterson *et al*. [30]. The mixture contained 0.135 mL of 20% TiCl_4_-dense HCl resolution, 0.1 mg/mL sample resolution, 0.185 mL of 0.17 M phosphate buffer solution (pH7.8) and 0.2 mL of 17.0 M NH_3_·H_2_O were incubated at room temperature for 5 min until a white floc appeared. The floc was dissolved with 3 mL of 3 M H_2_SO_4_ to test its absorbency (A_1_) at 410 nm. The samples were substituted by deionized water and repeated the procedures mentioned above to test the absorbency of blank (A_0_). The sample was substituted by Vc (0–100 μg/mL) and repeated the procedures mentioned above to get the standard curve. The hydrogen peroxide radical-scavenging activity was shown with Vc equivalent antioxidant capacity per 1 mg dry weight (μg Vc/mg DW).

### 3.8. Hydroxyl Radical-Scavenging Activity 

The tested method was optimized based on Rathee’s method [31] with some modifications. The mixture contained 0.2 mL of 10 mM FeSO_4_-EDTA solution, 0.5 mL of 10 mM D-deoxyribose solution, 0.1 mL of the different linear gradient concentrations sample solution and 1.8 ml of phosphate buffer solution. The mixtures were added to 0.2 mL of 10 mM H_2_O_2_, respectively, incubated at 37 °C for 1 h, and then added 1.0 mL of 2.8 % TCA solution and 10 mL of 1.0% TBA solution and incubated at 100 °C for 15 min. At last the absorbencies (A_s_) were tested at 532 nm after cooled fully. The procedures mentioned above was repeated except for being not added samples to test the absorbency of the blank (A_c_), and being not added samples and no incubation at 37 °C to test the absorbency of the blank (A_0_). Sodium benzoate was used as the positive control. The hydroxyl radical-scavenging effects of the samples were calculated respectively according to the following equation:

Hydroxyl radical-scavenging activity (%) = (A_c_ − A_s_) × 100/ (A_c_ − A_0_).

The linear regression equations of the hydroxyl radical-scavenging activity on the concentration of sample were established, and then the EC_50_ values were calculated in these equations.

### 3.9. Determination of the Amount of Total Phenolics

The amount of total phenolics was determined by a spectrophotometric method [19,32]. Briefly, sample fractions (1.0 mL) were was mixed with distilled water (9.0 mL) in a 25 mL volumetric flask. Then Folin-Ciocalteu phenol reagent (1.0 mL) was added to the mixture which was then shaken. The mixture was kept for 5 min, followed by the addition of 7% Na_2_CO_3_ solution (10 mL). The mixed solution was then diluted to 25 mL with distilled water and mixed thoroughly. After 90 min of reaction at room temperature, the absorbance versus a blank was measured at 750 nm. The standard curve for total phenolics was developed using gallic acid standard solution (0–100 mg/L) under the same procedure described above. The total phenolics of extract fractions were expressed as milligrams of gallic acid equivalents (GAE) per 100 g of dried sample.

### 3.10. Determination of the Amount of Total Flavonoids

The amount of total flavonoids was measured using the method described by Jia *et al*. [21,33]. Briefly, sample fractions or standard solution of rutin (1 mL) was mixed with distilled H_2_O (4 mL) in a 10 mL volumetric flask, followed by the addition of 5% NaNO_2_ solution (0.3 mL). After 5 min, 10% AlCl_3_ solution (0.3 mL) was added. At 6 min, 1 M NaOH solution (2 mL) was added to the mixture. Immediately, distilled H_2_O (2.4 mL) was added to the reaction flask and the contents mixed well. The absorbance versus a blank was measured at 510 nm. Measurements were calibrated to a standard curve of prepared rutin standard solution (0–0.5 mg/L). The total flavonoids of the extract fractions were expressed on an extract weight basis as mg/g rutin equivalents (RE). All samples were analyzed in three replications.

### 3.11. Statistical Analyses

Data were expressed as means ± standard deviation (S.D.) of three parallel measurements. Statistical calculations were carried out by SAS. Analysis of variance was performed by the ANOVA procedures. Duncan’s new multiple-range test was used to determine the difference of means. Analysis of regression was performed by the REG procedures.

## 4. Conclusions

This data presented in this paper indicates that the ethyl acetate fraction of papaya seed extract had the strongest antioxidant activity, and the *n*-butanol fraction had the second strongest antioxidant activity. The DPPH and the hydroxyl free radical-scavenging activities of the ethyl acetate fraction were stronger than those of ascorbic acid and sodium benzoate, respectively, indicating that the antioxidant components in papaya seeds mainly concentrated in the ethyl acetate fraction and the *n*-butanol fraction. The amount of total phenolics and total flavonoids in ethyl acetate fraction were the highest among all fractions, and that in *n*-butanol fraction took the second place. It is reported that *p*-hydroxybenzoic acid and vanillic acid which are widely found in fruits and vegetables have strong antioxidant activities [34,35,36,37,38]. Our results indicated that *p*-hydroxybenzoic acid and vanillic acid are the main constituents of the ethyl acetate fraction, accounting 75% of the total, so the two compounds contribute to the antioxidant activities of the ethyl acetate fraction from the seeds of papaya. Therefore, papaya seeds and these compounds might be used as natural antioxidants.

## Figures and Tables

**Table 1:** The regression equations of the DPPH radical scavenging rate on the sample concentration and the EC_50_ values.

Extract and fraction	The regression equations	Determined coefficients (r^2^)	F_(1,4)_-values	Pr	EC_50_ (μg/mL)
Ethanol	y = 0.1931x + 2.7601	0.9950	798.97	<0.0001	248.63
Petroleum ether	y = 0.0443x + 9.7114	0.9110	40.93	0.0031	1009.50
Ethyl acetate	y = 0.5138x + 17.0600	0.9860	281.15	<0.0001	64.61
*n*-Butanol	y = 0.3263x + 13.9250	0.9815	212.06	0.0001	109.30
Water	y = 0.0256x + 1.1455	0.7767	13.92	0.0203	1628.33
Ascorbic acid	y = 0.5742x + 11.8220	0.9660	113.52	0.0003	66.96

**Table 2:** The performances of the antioxidant activities, the O_2_^−^ and H_2_O_2_ radical scavenging activities.

Extract and fraction	The TEAC value (mmolTrolox/g DW)	The antioxidant activity by FRAP assay (μmol FeSO_4_/g DW)	The O_2_^−^ radical scavenging activity (μmol α-Tocopherol/g DW)	The H_2_O_2_ radical scavenging activity (μg Vc/mg DW)
Petroleum ether	1.06 ± 0.04C	828.33 ± 10.4083C	1151.79 ± 60.21B	68.09 ± 5.56B
Ethyl acetate	2.48 ± 0.42B	1116.67 ± 7.6376A	1318.73 ± 19.52A	73.38 ± 6.01AB
*n*-Butanol	4.75 ± 0.66A	993.33 ± 65.2559B	1365.86 ± 94.64A	79.24 ± 4.54A
Water	0.29 ± 0.04D	998.33 ± 5.7735B	242.06 ± 8.21D	12.74 ± 0.93D
Ethnol	2.08 ± 0.27B	1026.67 ± 17.5594B	947.05 ± 39.15C	48.91 ± 2.26C

*Note*: The numbers followed with the different capital letters showed the significance level at 0.01, and followed with the same capital letters showed the insignificant differences at the significance level of 0.01. The same comment applies to Table 4.

**Table 3:** The regression equations of the hydroxyl-radical scavenging rate on the sample concentration and the EC_50_ values.

Extract and fraction	The regression equations	Determinated coefficients (r^2^)	F_(1,4)_-values	Pr	EC_50_ (μg/mL)
Ethanol	y = 60.5610x + 4.1270	0.9972	1433.99	<0.0001	0.7575
Petroleum ether	y = 2.8440x + 35.2600	0.2735	1.50	0.2872	5.1828
Ethyl acetate	y = 42.4940x + 45.9880	0.9869	302.34	<0.0001	0.0944
n-Butanol	y = 45.5240x + 40.4340	0.9668	116.51	0.0004	0.2101
Water	y = 27.7650x + 40.9200	0.7327	11.25	0.0285	0.3270
Sodium benzoate	y = 173.5800x + 33.4850	0.9470	71.52	0.0011	0.0951

**Table 4:** The amount of the total phenolics and the total flavonoids in fractions of papaya seeds.

Extract and fraction	Phenolics (mg GAE/100 gDW)	Flavonoids (mg RE/g DW)
Petroleum ether	522.67 ± 94.39D	–
Ethyl acetate	1945.48 ± 45.55A	117.48 ± 15.54A
n-Butanol	832.25 ± 125.91C	32.04 ± 2.45B
Water	276.64 ± 47.53E	4.22 ± 0.14C
Ethanol	1132.41 ± 162.58B	22.47 ± 0.69B
